# Systemic Reprogramming of Monocytes in Cancer

**DOI:** 10.3389/fonc.2020.01399

**Published:** 2020-09-17

**Authors:** Máté Kiss, Aarushi Audhut Caro, Geert Raes, Damya Laoui

**Affiliations:** ^1^Myeloid Cell Immunology Lab, VIB Center for Inflammation Research, Brussels, Belgium; ^2^Lab of Cellular and Molecular Immunology, Vrije Universiteit Brussel, Brussels, Belgium

**Keywords:** classical monocyte, cancer, tumor, monocyte reprogramming, tumor-educated monocytes, hematopoiesis, monopoiesis, peripheral blood

## Abstract

Monocytes influence multiple aspects of tumor progression, including antitumor immunity, angiogenesis, and metastasis, primarily by infiltrating tumors, and differentiating into tumor-associated macrophages. Emerging evidence suggests that the tumor-induced systemic environment influences the development and phenotype of monocytes before their arrival to the tumor site. As a result, circulating monocytes show functional alterations in cancer, such as the acquisition of immunosuppressive activity and reduced responsiveness to inflammatory stimuli. In this review, we summarize available evidence about cancer-induced changes in monopoiesis and its impact on the abundance and function of monocytes in the periphery. In addition, we describe the phenotypical alterations observed in tumor-educated peripheral blood monocytes and highlight crucial gaps in our knowledge about additional cellular functions that may be affected based on transcriptomic studies. We also highlight emerging therapeutic strategies that aim to reverse cancer-induced changes in monopoiesis and peripheral monocytes to inhibit tumor progression and improve therapy responses. Overall, we suggest that an in-depth understanding of systemic monocyte reprogramming will have implications for cancer immunotherapy and the development of clinical biomarkers.

## Introduction

Monocytes are the third most abundant immune cell population in the peripheral blood after neutrophils and lymphocytes, representing ~4–11% of leukocytes in the circulation in humans and 1–5% in mice (1, 2). Based on the expression of surface markers, size, morphology, location in the blood vessel, and functionality, two major monocyte subsets can be distinguished both in human and mouse. Classical monocytes (CD14^+^CD16^−^CCR2^+^CX3CR1^low^HLA-DR^+^ in human, Ly6C^high^CCR2^+^CD43^−^CX3CR1^low^MHC-II^−^ in mouse, after exclusion of lymphoid cells and granulocytes) are large (10–14 μm diameter in mouse) granular cells whose primary function is to extravasate and differentiate into macrophages upon tissue injury and, in certain tissues, replenish tissue-resident macrophages in homeostasis. Non-classical monocytes (CD14^−^CD16^+^CCR2^−^CX3CR1^high^HLA-DR^+^ in human, Ly6C^low^CCR2^−^CD43^+^CX3CR1^high^MHC-II^−^ in mouse) are smaller (8–12 μm diameter in mouse) less granular cells which crawl along vessels and scavenge the luminal surface to maintain endothelial integrity (3–7). Non-classical monocytes differentiate from classical monocytes in the circulation that is triggered by signals from the vascular endothelium (8, 9). Accordingly, a continuum of intermediate cell states between the two subsets exists which was revealed by single-cell RNA sequencing (scRNAseq) in both human and mouse (9, 10). The majority of classical monocytes leave the circulation within 1 day and extravasate into tissues to replenish macrophages while only a small fraction of them differentiates into non-classical monocytes to remain in the circulation for several days (11, 12).

Classical monocytes, classical monocyte-derived tumor-associated macrophages, and non-classical monocytes have been extensively described to influence tumor progression through regulating cancer cell survival, antitumor immunity, angiogenesis, and metastasis. The mechanistic details of these activities have been reviewed elsewhere (13–18). Much less is known about whether tumors remotely induce alterations in monopoiesis and circulating monocytes. In this review we summarize evidence for altered classical monocyte abundance and phenotype in cancer and we discuss the potential implications of this phenomenon for tumor progression. Due to their shared ontogeny, the phenotype of non-classical monocytes is likely to be affected by cancer as well, however, evidence for this remains scarce.

## Monopoiesis in Cancer

Elevated peripheral blood monocyte counts in cancer have been described in both humans and mice (19–21). Patients with higher blood monocyte counts reportedly have a worse disease prognosis in several cancer types (20, 22–26). Consistent with the notion that classical monocytes can give rise to tumor-associated macrophages, blood monocyte counts correlate with the abundance of macrophages infiltrating prostate tumors, however, more studies are needed to establish whether such correlation is a general phenomenon (23). Elevated monocyte levels can be caused either by enhanced mobilization from the bone marrow or increased monopoiesis, both of which have been observed in cancer. CCL2, the central regulator of monocyte mobilization from the bone marrow, often shows higher serum levels in both mouse and human cancer (27–31). Accordingly, elevated peripheral blood monocyte levels in pancreatic cancer patients were associated with reduced monocyte abundance in the bone marrow, suggesting their enhanced egress (20).

**Figure 1:**
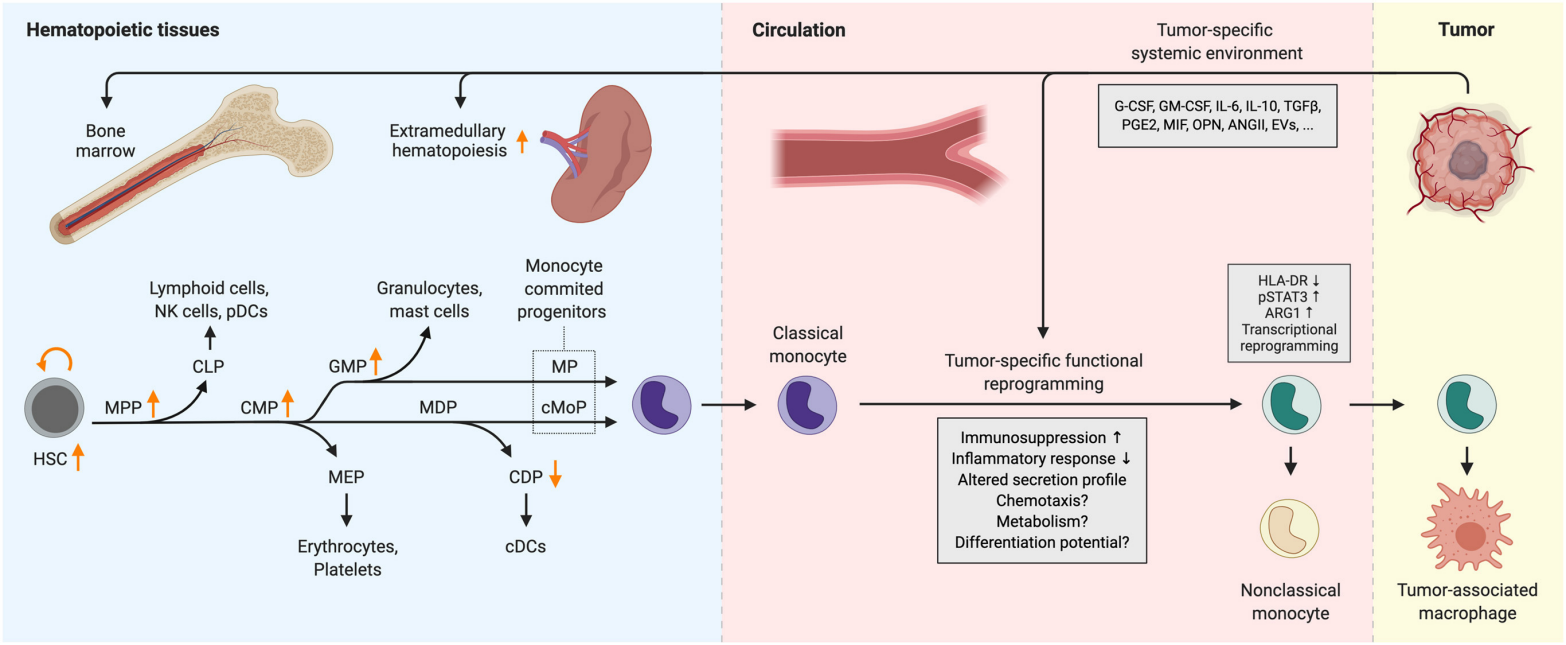
Cancer-induced reprogramming of monopoiesis and circulating monocytes. Arrows indicate changes in the abundance of progenitor populations in cancer. Functional alterations that have not been characterized extensively are indicated with question marks. ANGII, angiotensin II; ARG1, arginase 1; cDC, conventional dendritic cell; CDP, common dendritic cell progenitor; CLP, common lymphoid progenitor; cMoP, common monocyte progenitor; CMP, common myeloid progenitor; EVs, extracellular vesicles; G-CSF, granulocyte colony-stimulating factor; GM-CSF, granulocyte-macrophage colony-stimulating factor; GMP, granulocyte-monocyte progenitor; HLA-DR, human leukocyte antigen DR; HSC, hematopoietic stem cell; IL-6/10, interleukin-6/10; MDP, monocyte-dendritic cell progenitor; MEP, megakaryocyte-erythrocyte progenitor; MIF, macrophage migration inhibitory factor; MP, monocyte progenitor; MPP, multipotent progenitor; NK cells, natural killer cells; OPN, osteopontin; pDC, plasmacytoid dendritic cell; PGE2, prostaglandin E2; pSTAT3, phosphorylated signal transducer and activator of transcription 3; TGFβ, transforming growth factor β.

Emerging evidence indicates that tumors also remotely influence hematopoiesis. In the steady-state, monocytes are produced in the bone marrow by hematopoietic stem cells (HSCs) which give rise to progenitors with progressively restricted lineage potential ultimately resulting in the generation of monocyte-committed progenitors (Figure 1). HSCs self-renew and generate multipotent progenitors (MPPs), which further differentiate into common myeloid progenitors (CMPs), and common lymphoid progenitors (CLPs). CMPs have the capacity to differentiate into megakaryocyte and erythrocyte progenitors (MEPs) and granulocyte and macrophage progenitors, also known as granulocyte-monocyte progenitors (GMPs). Within the GMP population, monocyte-dendritic cell progenitors (MDPs) emerge which can only give rise to conventional dendritic cell progenitors (CDPs) and common monocyte progenitors (cMoPs), the latter giving rise to classical monocytes (7, 32). Notably, recent research shows that MDPs can develop directly from CMPs (33). GMPs can also generate classical monocytes through a monocyte progenitor (MP) and these monocytes retain a transcriptional profile distinct from their MDP-derived counterparts, characterized by the expression of several neutrophil-associated genes (33, 34). These “neutrophil-like” monocytes have been detected via scRNAseq in the blood and tumors of humans and mice with non-small cell lung cancer, however, it remains unknown whether their distinct transcriptional profile endows them with unique functional characteristics (35).

Gradual commitment to the monocyte lineage is determined by the relative activity of key transcription factors in hematopoietic progenitors [reviewed in (32, 36)]. Monocyte and macrophage development are critically dependent on PU.1, whose expression increases from the CMP stage and acts as a pioneer transcription factor to bind closed chromatin and cooperate with other myeloid transcription factors in order to activate a myeloid lineage specific transcriptional program. A key growth factor in monocyte and macrophage development is M-CSF (also known as CSF-1) which not only promotes survival and proliferation of myeloid progenitors, but also instructs the commitment of GMPs toward monocytic cells rather than granulocytes (37, 38). In addition, M-CSF can directly induce PU.1 in HSCs, instructing early commitment toward the myeloid lineage (39). According to the current model, PU.1 induces IRF8 expression in MDPs which further promotes monocyte/dendritic cell over granulocyte differentiation potential in progenitors (36, 40). IRF8 forms a heterodimer with PU.1 and induces the expression of the transcription factor KLF4, which is indispensable for the acquisition of a transcriptional program endowing mature monocyte identity (41–43). The C/EBP transcription factors also play key roles in both monocyte and granulocyte development. C/EBPα is essential for steady-state granulopoiesis and the relative activity of PU.1 and C/EBPα in GMPs is a critical determinant of monocyte/macrophage vs. neutrophil cell fate (44, 45). C/EBPβ is not only required for emergency granulopoiesis in response to cytokines, but also supports the survival of monocytes in the periphery (46, 47).

With the emergence of single-cell resolution transcriptomics and fate-mapping technologies, the hierarchical lineage tree model of hematopoiesis is being replaced by a lineage continuum model in which the progenitor populations defined above are rather snapshots of a continuum and encompass a transcriptionally diverse mixture of cells with different degrees of fate commitment (48). In fact, lineage-committed precursors have been found in progenitor populations which have been previously defined as multipotent (48). Nevertheless, progenitor populations defined by well-established surface markers provide a useful framework to understand hematopoiesis and evaluate its quantitative and qualitative alterations in disease.

Cancer is often accompanied by elevated serum levels of cytokines that are involved in controlling hematopoiesis, including KITLG, G-CSF, GM-CSF, and M-CSF (49–54). Enhanced production of these cytokines can be a result of malignant transformation of cells and can therefore be dictated by the genetic alterations that occur during tumor progression (55–59). Altered production of these factors together with the low-grade systemic inflammation often associated with tumor development leads to remote reprogramming of myelopoiesis (60). This is characterized by the expansion of HSC, myeloid-skewed MPP, CMP, and GMP, but not CLP and MEP populations, indicating a tumor-induced myeloid bias in hematopoiesis (61–64) (Figure 1). Myeloid expansion in the bone marrow was driven by G-CSF in the MMTV-PyMT mouse model of breast carcinoma, while it was found to be TNFα-dependent in Lewis lung carcinoma and MC57 fibrosarcoma (61, 62). Similarly, the frequency of HSC, MPP, and GMP populations are elevated in the peripheral blood of patients with various types of solid tumors (52). Conversely, the abundance of the CDP population decreases in both breast and pancreatic cancer patients while the MDP population remains largely unaffected due to the inhibitory effect of G-CSF on the differentiation potential of MDP toward CDP (51) (Figure 1). G-CSF and GM-CSF suppress IRF8 expression via STAT3 and STAT5, respectively, thereby skewing myelopoiesis toward granulocyte progenitors (65–67). Due to elevated systemic levels of G-CSF and GM-CSF, this can occur in cancer. Accordingly, MDPs from breast tumor-bearing mice showed higher levels of phosphorylated STAT3 and lower IRF8 expression (51). Expansion of GMPs in response to G-CSF in cancer may not only drive the production of granulocytes, but also monocytes, as the tumor-induced expansion of circulating monocytes is completely abrogated in G-CSF receptor-deficient mice (68). Similarly, GM-CSF treatment in mice increased the abundance of both monocytes and neutrophils in the bone marrow (54). Analogously, administration of an antitumor vaccine containing GM-CSF led to the expansion of immunosuppressive monocytes in melanoma patients (69). Indeed, GM-CSF treatment of human HSCs *in vitro* results in the generation of CD14^+^HLA-DR^−^PD-L1^+^ monocyte-like cells which are highly immunosuppressive and this effect was augmented by the addition of IL-6 or TGFβ (52, 54, 70, 71). Combination of G-CSF+GM-CSF or G-CSF+IL-6 treatment of HSCs generates similar immunosuppressive cells, however, these also upregulate arginase-1, an enzyme that catabolizes L-arginine, an amino acid essential for T-cell proliferation (52, 70, 72).

Cancer not only reprograms hematopoiesis in the bone marrow but also supports expansion of myelopoiesis in extramedullary sites, primarily in the spleen. HSC, CMP, GMP, and MDP populations greatly expand in the splenic red pulp of tumor-bearing mice and cancer patients due to recruitment of progenitors from the circulation followed by local proliferation (21, 73–75). In homeostasis, tissue-migratory hematopoietic progenitors eventually return to the circulation through the lymphatic system, which is driven by sphingosine-1 phosphate (S1P) gradients (76). However, in murine lung cancer this process is perturbed as tumor cell-derived angiotensin II released to the circulation causes downregulation of S1P receptor 1 on hematopoietic progenitors, leading to their retention, and accumulation in the spleen (77). In addition, increased CCL2 production by splenic myeloid cells and stromal cells in cancer appears to contribute to the accumulation of myeloid progenitors, which upregulate their CCR2 expression in the spleen (73, 75). Proliferation of splenic myeloid progenitors is also supported by tumor cell-derived osteopontin (78).

In some mouse tumor models, monocytes isolated from the spleen have been shown to suppress T-cell activation via nitric oxide production, which mainly interferes with IL-2 receptor signaling (72, 79). For this reason, these cells were termed monocytic myeloid-derived suppressor cells (Mo-MDSC), a term still used to denote immunosuppressive monocytes, albeit the surface marker expression of these cells in many cases closely resembles classical monocytes (79). Accordingly, bone marrow HSC transferred into spleens gave rise to T-cell suppressive myeloid cells in tumor-bearing mice but not in healthy mice (73). These results suggest that the cancer-conditioned splenic tissue niche can skew monopoiesis toward the generation of immunosuppressive monocytes. Consistent with the notion that splenic monocytes undergo extensive reprogramming in cancer, scRNAseq analysis of splenic monocytes revealed tumor-induced expansion of a distinct monocyte state in mouse breast cancer (80). Splenic monocytes from breast tumor-bearing mice showed more than 200 differentially expressed genes compared to healthy mice, including the upregulation of genes involved in the promotion of inflammation (*Il1b, Saa3, Junb*), angiogenesis (*Prok2*), chemotaxis (*Ccr1, Cxcr2*), and antiviral response (*Ifitm1*) (80). Two key factors driving the reprogramming of progenitors in the spleen appear to be GM-CSF and IL-6. Splenic stromal cells upregulate IL-6 in mice with hepatocellular carcinoma, which drives autocrine GM-CSF production by splenic HSCs and this interaction appears to be critical for the generation of immunosuppressive progeny (73). In line with these findings, GM-CSF treatment in mice increased the abundance of Ly6C^high^ monocytes in the spleen (54). Similarly, low levels of GM-CSF are sufficient to induce nitric oxide synthase in bone marrow-derived Ly6C^high^ murine monocytes and render them strongly T-cell suppressive (54). Notably, the impact of GM-CSF on monocytes is likely to be dependent on their developmental stage at the time of exposure as well as the tissue context (81). GM-CSF secreted by activated T-cells has been shown to induce a pro-inflammatory monocyte phenotype in experimental autoimmune encephalitis (82). Some studies suggest that GM-CSF-dependent monocyte activation during chimeric antigen receptor T-cell therapy can contribute to the development of potentially fatal treatment-related toxicities, including cytokine release syndrome and neuroinflammation (83, 84). The immunostimulatory activity of GM-CSF provided a basis for its use as an adjuvant in anticancer vaccines (81, 85–87). However, GM-CSF-containing vaccine formulations may not only promote antitumor immunity, but in some cases also cause the emergence of immunosuppressive monocytes in the circulation, as mentioned above (69).

## Cancer-Induced Phenotypical Alterations in Circulating Monocytes

The distant tumor not only skews the differentiation path of myeloid progenitors in hematopoietic tissues but also influences the phenotype of circulating monocytes (Figure 1). The most widely reported cancer-induced phenotypical alteration in human peripheral blood monocytes is the acquisition of immunosuppressive activity (19, 69). This generally coincides with the downregulation of the MHC class II surface protein HLA-DR, a key mediator of antigen presentation which is highly expressed on monocytes in healthy individuals. Additional surface marker changes have also been reported, including the downregulation of CD86 (88–90) and upregulation of IL4Rα (91, 92) and TIE2 (93). On the basis of their immunosuppressive activity, CD14^+^HLA-DR^low^ monocytes are often referred to as M(o)-MDSCs, analogous to T-cell suppressive mouse monocytes isolated from the spleen of tumor-bearing mice. Interestingly, a similar HLA-DR^low^ monocyte phenotype has been observed in patients with sepsis and the transcriptional signatures of monocytes in sepsis and metastatic cancer show remarkable similarity (88, 94, 95).

One of the major mechanisms responsible for the immunosuppressive activity of monocytes in cancer patients appears to be their elevated arginase-1 expression and activity which restricts the amount of L-arginine available for T-cells (19, 96–98). Accordingly, inhibition of arginase-1 or increasing the amount of available L-arginine decreases their T-cell suppressive effect *in vitro* (91, 97–99). Other mechanisms that may be responsible for the immunosuppressive activity include upregulation of PD-L1 or GPNMB and production of TGFβ or reactive oxygen species (69, 91, 100–102).

The frequency of CD14^+^HLA-DR^low^ monocytes has been shown to increase with tumor stage and correlate with poor survival in many different solid tumor types [reviewed in (103, 104)]. In accordance with their immunosuppressive effect, high levels of CD14^+^HLA-DR^low^ monocytes are associated with significantly lower levels of tumor-specific T-cells in the circulation of cancer patients (105). In addition, patients with low pretreatment levels of CD14^+^HLA-DR^low^ monocytes are more likely to respond to immune checkpoint blockade therapy, providing a rationale to use pretreatment HLA-DR^low^ monocyte frequency as a predictive biomarker for therapy response (106–109). Intriguingly, patients who responded to anti-CTLA4 immune checkpoint blockade showed a progressive reduction in the frequency of CD14^+^HLA-DR^low^ monocytes following treatment, in contrast to non-responders (110, 111).

While the emergence of immunosuppressive activity in cancer-educated monocytes has been widely observed, alterations in their cytokine secretion appear to be more variable across different cancer types. CD14^+^HLA-DR^low^ monocytes from melanoma patients showed increased secretion of TGFβ (69, 102), however, this was not observed in other studies in melanoma and breast cancer (88, 91). Monocytes from breast cancer patients secreted lower levels of IL-1β, IL-6, and TNF (88), while monocytes from renal cell carcinoma patients showed elevated production of these cytokines along with IL-10, CCL3, IL-8, and VEGFα (112). In the latter study, these changes led to an enhanced ability to promote angiogenesis and cancer cell invasion *in vitro* that was dependent on the secretion of VEGFα and matrix metalloproteinases, respectively, (112). In contrast, *VEGFA* expression was downregulated in monocytes of breast cancer and melanoma patients (113, 114).

Classical monocytes isolated from breast cancer patients also exhibit altered response to inflammatory stimuli, as indicated by their impaired secretion of TNFα and IL-1β in response to bacterial lipopolysaccharide (88, 115). In addition, classical monocytes from lymphoma and breast cancer patients showed reduced responsiveness to IFNγ as indicated by the lower levels of STAT1 phosphorylation following stimulation (98, 116). Remarkably, breast cancer patients with a low monocyte IFNγ response were significantly more likely to relapse (116). Hence, the level of IFNγ-induced STAT1 phosphorylation in peripheral blood monocytes at diagnosis could be used as a prognostic biomarker for relapse-free survival independent of other clinicopathologic characteristics (116).

Transcriptomic analyses in peripheral blood monocytes from cancer patients vs. healthy donors revealed extensive cancer-induced transcriptional changes and provided several important lessons about monocyte reprogramming in cancer (Table 1).

**Table 1:** Summary of studies comparing the transcriptome of peripheral blood monocytes from cancer patients and healthy individuals.

**Cancer type**	**Number of patients**	**Markers used for monocyte isolation**	**Method**	**Publication**	**Accession number**
Breast cancer (metastatic)	Healthy: 3 Cancer: 4	CD14^+^CD16^−^ (MACS)	Microarray	(88)	GSE65517
Breast cancer	Healthy: 8 Cancer: 8	CD14^+^HLA-DR^+^ (FACS)	Microarray	(115)	NA
Breast and endometrial cancer	Healthy: 45 Cancer: 32 (breast), 3 (endometrial)	Lin^−^CD45^+^CD11b^+^CD14^+^ (FACS)	RNA-seq	(113)	GSE117970
Colorectal carcinoma	Healthy: 38 Cancer: 55	CD14^+^ (MACS)	Microarray	(117)	GSE47756
Colorectal carcinoma (metastatic)	Healthy: 20 Cancer: 3	CD14^+^ (MACS)	RNA-seq	(95)	GSE133822
Glioblastoma	Healthy: 4 Cancer: 4	CD14^+^ (MACS)	Microarray	(118)	GSE77043
Melanoma	Healthy: 4 Cancer: 4	Lin^−^CD14^+^CD16^−^HLA-DR^+^ (FACS)	RNA-seq	(114)	E-MTAB-6214
Pancreatic ductal adenocarcinoma	Healthy: 3 Cancer: 5	CD14^+^CD16^−^ (FACS)	Microarray	NA	GSE60601
Pancreatic ductal adenocarcinoma	Healthy: 9 (from public datasets) Cancer: 7	CD14^+^ (MACS)	Microarray	(19)	NA
Renal cell carcinoma	Healthy: 4 Cancer: 4	CD14^+^ (MACS)	Microarray	(112)	GSE38424

*MACS, magnetic cell separation; FACS, fluorescence-activated cell sorting*.

Firstly, by utilizing classification algorithms, the cancer-induced gene signature in blood monocytes can be used as a diagnostic biomarker. The first proof-of-concept studies testing cancer detection based on transcriptional alterations in peripheral blood monocytes demonstrated 93–100% sensitivity (i.e., the proportion of cancer patients that are correctly identified as such), albeit somewhat more limited 69–93% specificity (i.e., the proportion of healthy patients that are correctly identified as such) (113, 117).

Secondly, the gene expression changes in monocytes induced by distinct cancer types show remarkably little overlap. Gastric cancer and pancreatic cancer failed to induce the gene expression signature which was identified in colon cancer (117). Similarly, the transcriptomic changes induced by endometrial and breast cancer assessed in the same study showed <50% overlap (113).

Furthermore, the cancer-induced transcriptional profiles show considerable interpatient heterogeneity within a given cancer type, uncovering patient subsets with differential reprogramming (19, 115). Specifically, greatly differing monocyte reprogramming (>1,000 differentially expressed genes) could be observed between pancreatic cancer patients in which immunosuppressive monocytes emerged and patients whose monocytes remained non-suppressive (19). Among breast cancer patients, differential responsiveness of monocytes to IFNγ+GM-CSF stimulation was associated with distinct gene expression profiles, including differential expression of genes linked to the IFN response (115).

Finally, transcriptional profiling of monocytes from colorectal cancer patients revealed that monocyte reprogramming not only occurs after systemic dissemination of cancer, but also in patients with localized early stage tumors (117).

Transcriptomic analyses provided indications that monocytes may exhibit additional phenotypical alterations. Several studies have shown changes in the expression of genes involved in cell adhesion, migration, and chemotaxis, such as elevated *CCR2* and *CX3CR1* expression (112–115). Accordingly, classical monocytes from non-small cell lung cancer patients showed enhanced migration toward cancer cells, however, the underlying mechanisms remain to be determined (96). Intriguingly, multiple studies from breast cancer patients showed the downregulation of *HIF1A* expression in monocytes, suggesting that cancer may impair their response to hypoxia (88, 113, 115).

Monocytes also exhibited cancer-induced changes in the expression of numerous metabolic genes in several tumor types (19, 114, 115). Namely, immunosuppressive monocytes in pancreatic cancer showed upregulation of genes involved in fatty acid and lipoprotein metabolism (*CD36, LYPLA1, CERS5*) ATP metabolism (*ATP5F1C, ATP5MC2, SDHB*), glucose metabolism (*PDK4, GXYLT1*), and amino acid metabolism (*ERICH1, GLS, CTSC, ARG1, NAT2, UST, OXR1*) when compared to non-immunosuppressive monocytes (19). Similarly, monocytes from breast cancer and glioblastoma patients showed altered expression of genes involved in oxidative phosphorylation and fatty acid metabolism (115, 118). Monocytes from melanoma patients showed downregulation of several nutrient transporters, including the glucose transporter *SLC2A3* and the amino acid transporters *SLC7A5, SLC7A11, SLC3A2* (114). It remains to be elucidated whether these gene expression changes have an impact on cellular metabolism.

Tumor-induced reprogramming may also impair the ability of monocytes to initiate a physiological differentiation program upon tissue infiltration. Monocytes from breast cancer patients showed reduced expression of *ID2* and *MAFB*, transcriptional regulators playing key roles in dendritic cell and macrophage differentiation, respectively (113, 115, 119, 120). In line with these data, dendritic cells differentiated from monocytes of breast cancer patients *in vitro* showed a reduced capacity to stimulate T-cell proliferation and induced a higher number of regulatory T-cells compared to healthy controls (121).

It is difficult to determine whether phenotypical changes observed in circulating monocytes stem from alterations in hematopoietic progenitors or they are mainly acquired in the circulation. Certainly, elevated secretion of cytokines, such as G-CSF, GM-CSF, and IL-6 in cancer can favor the development of monocytes with an altered phenotype in the bone marrow and spleen, as described above. However, monocyte reprogramming has been observed in patients in the absence of emergency myelopoiesis, indicating that reprogramming of mature monocytes in the circulation probably also occurs (88, 117). Indeed, co-culture with cancer cells or treatment with cancer cell supernatants can induce transcriptional changes and phenotypical alterations in mature monocytes from healthy individuals, including the induction of immunosuppressive, proinvasive, and proangiogenic phenotypes (68, 112, 117, 122, 123). Extracellular vesicles (EVs) released from cancer cells may be important in relaying reprogramming signals as they were found to be sufficient to induce immunosuppressive activity in healthy monocytes *in vitro* (102, 124–128). One of the mechanisms responsible for this appears to be the activation of Toll-like receptors (TLR) on monocytes by heat-shock proteins (HSP) expressed on the surface of EVs, such as HSP72 and HSP86, activating TLR2 and TLR4, respectively (126, 127). Additional factors that have demonstrated a reprogramming effect on healthy monocytes include IL-10, MIF, and prostaglandin E2 (PGE2), which may also be produced by non-malignant cells (122, 123, 129–132).

Transcriptional reprogramming of monocytes is likely to be driven by the activation of a distinct set of transcription factors dictated by the tumor/patient-specific systemic environment. The most studied example is the acquisition of immunosuppressive activity, which, in many cases, is driven by the transcription factor STAT3. Co-culture of healthy monocytes with cancer cells or treatment with cancer cell-derived EVs induces STAT3 activation (68, 126). Correspondingly, suppressive monocytes from cancer patients show elevated levels of phosphorylated STAT3, and inhibition of STAT3 reverses the arginase-dependent immunosuppressive activity (19, 91, 99).

It is currently unclear whether tumor removal leads to the complete reversal of monocyte phenotype to a healthy state. Diminished HLA-DR expression on monocytes from glioblastoma patients returned to normal levels 8 days after tumor removal (133). Similarly, surgical removal of colorectal tumors led to the reversal of a previously upregulated gene set to levels comparable to healthy individuals (117). In contrast, the frequency of HLA-DR^low^ classical monocytes in prostate and colorectal cancer patients did not return to healthy levels 1 month after surgery, suggesting that, in some cases, alterations may persist after curative treatment (134).

## Therapeutic Implications

Understanding how monocytes respond to cancer will pave the way toward targeted treatments that can interfere with the cellular pathways mediating tumor-induced functional alterations. The best characterized example of such therapeutic strategy is the prevention of cancer-induced monocytosis via inhibiting the CCL2-CCR2 chemokine axis (17). A small-molecule CCR2 inhibitor has been already tested in a phase I clinical trial and proved effective in reducing peripheral monocyte numbers, thereby decreasing the abundance of tumor-associated macrophages in pancreatic cancer (135). This was associated with increased T-cell infiltration and elevated expression of immunostimulatory factors in tumors, indicating a better antitumor immune response, and warranting further clinical studies (135). Identification of angiotensin II as a crucial regulator of cancer-induced extramedullary hematopoiesis raised the question whether angiotensin-converting enzyme (ACE) inhibitors, widely used to treat hypertension, could suppress heightened extramedullary monocyte production and subsequent macrophage accumulation in tumors (77). Indeed, the ACE inhibitor enalapril was able to suppress splenic monopoiesis, reduce the number of tumor-associated macrophages and provided a survival benefit to mice with lung tumors (77). In a mouse model of hepatocellular carcinoma, the multikinase inhibitor sorafenib has been reported to similarly reduce splenic hematopoiesis presumably by inhibiting c-Kit (73). Although sorafenib alone did not prolong survival, it enhanced the therapeutic efficacy of anti-PD-L1 immune checkpoint blockade (73). As mentioned above, GM-CSF promotes monocyte production both in the bone marrow and in the spleen. Accordingly, GM-CSF blockade in mice inhibited tumor-induced mobilization of CD11b^+^Gr1^+^ myeloid cells, resulting in enhanced antitumor T-cell responses (136, 137). The CD11b^+^Gr1^+^ cell subset comprises a heterogenous mixture of monocytes and granulocytes, therefore determining the impact of GM-CSF neutralization specifically on monocytes will require further investigation.

Among the factors mediating tumor-induced reprogramming of monocytes, PGE2 appears to be a promising candidate for therapeutic targeting. A PGE2 receptor 2 (EP2) antagonist (AH6809) prevented PGE2-induced NF-κB activation and subsequent *Nos2* expression in splenic and tumor-infiltrating monocytes, reducing their immunosuppressive activity and leading to an enhanced antitumor T-cell response in mouse models (132).

Tumor-induced alterations of kinase activity in monocytes are also an area of emerging interest and a promising therapeutic avenue considering the wide range of small-molecule kinase inhibitors available. In a mouse model of melanoma, splenic monocytes have been shown to upregulate the TAM receptor tyrosine kinases Axl, Mertk, and Tyro3, while circulating monocytes upregulated Mertk and Tyro3 (138). Targeting these kinases via the administration of a pan-TAM kinase inhibitor (UNC4241) was able to reduce the immunosuppressive activity of monocytes and enhance antitumor immunity (138). TAM kinases were suggested to promote serine phosphorylation of STAT3, leading to the activation of genes involved in immunosuppression, like *Nos2* and *Arg1* (138).

Besides TAM kinases, several additional reprogramming stimuli, such as IL-6, IL-10, and G-CSF, converge to STAT3 activation, making it another attractive therapeutic target. STAT3 inhibition via small-molecule inhibitors (e.g., CPA-7, JSI-124) has proved effective in eliciting antitumor immunity in mice, however, its effects on monocytes have not been characterized (139, 140). To avoid unwanted side-effects due to its pleiotropic role, therapeutic inhibition of STAT3 may require cell-specific targeting strategies. A potential approach to achieve this has been developed by linking a STAT3-targeting small interfering RNA or antisense oligonucleotide to a TLR9 agonistic CpG oligonucleotide which reportedly reduces *Stat3* expression specifically in TLR9-expressing myeloid cells while exerting an immunostimulatory effect via TLR9 activation (141, 142). This strategy has proved effective in boosting the antitumor immune response in several mouse models, however, it remains to be determined whether it efficiently targets monocytes and could reverse tumor-induced reprogramming (141–143).

In addition, inhibition of arginase-1 to alleviate monocyte/macrophage-mediated arginine-depletion and consequential immunosuppression may represent a potential therapeutic approach. To this end, a small-molecule arginase inhibitor (CB-1158) has been developed which elevated plasma and tumor arginine levels and enhanced antitumor T-cell and natural killer cell responses in mouse models (144). Remarkably, arginase inhibition also improved response to immune checkpoint blockade and adoptive T-cell therapy in several tumor models which are resistant to these treatments (144).

Therapies inducing systemic immune activation may also be able to reprogram monocytes before their arrival to tumors that is likely to influence their activity upon tumor infiltration. Administration of an agonistic anti-CD40 antibody led to systemic release of IFNγ, resulting in enhanced STAT1 activation in circulating monocytes (145). Recruitment of these pre-activated Ly6C^high^ monocytes was critically required for the anti-fibrotic activity of anti-CD40 therapy in pancreatic cancer (145). The elevated matrix metalloproteinase activity of recruited monocytes following treatment degraded the dense extracellular matrix of chemoresistant pancreatic tumors, rendering them responsive to gemcitabine therapy (145). It remains to be further characterized whether CD40 agonists or other immunostimulatory agents (e.g., TLR and STING agonists) are able to reprogram the transcriptome and phenotype of extratumoral monocytes either directly or indirectly, and how this impacts their activity and differentiation trajectory following extravasation.

## Conclusions and Perspectives

Recent studies have revealed that phenotypical alterations in peripheral blood monocytes can serve as diagnostic (113, 117), predictive (108), and prognostic (116) biomarkers. As monocytes can be easily obtained via blood sampling, these findings offer promising new tools for clinical oncology. The emergence of immunosuppressive monocytes in the circulation of cancer patients and their widely documented association with poor prognosis strongly suggest that cancer-induced monocyte reprogramming has an important role in tumor progression. Nevertheless, our understanding about the driving mechanisms of this phenomenon are far from complete. Transcriptomic analyses of circulating monocytes revealed that different cancer types induce distinct gene signatures and these transcriptional changes extend beyond the induction of an immunosuppressive phenotype. These studies showed that cancer also alters the expression of genes involved in a number of additional cellular functions, such as chemotaxis, metabolism, and differentiation, among others. Further studies are needed to confirm whether these transcriptional changes lead to functional reprogramming that may influence monocyte behavior.

Ultimately, the majority of circulating classical monocytes extravasate to replenish macrophages in tissues. This raises the question whether some of the cancer-induced changes in monocytes persist during the differentiation process and leave a mark on their progeny, thus causing a ripple effect on systemic immunity through altering the function of tissue macrophages. Accordingly, therapies which can pre-activate circulating monocytes may have the potential to skew their differentiation toward cytotoxic and/or T-cell stimulatory macrophages upon extravasation in the tumor. Identifying therapies capable of “re-educating” circulating monocytes will likely represent a useful strategy to prevent metastasis as rapid monocyte recruitment and differentiation into metastasis-supporting macrophages is increasingly appreciated as an important determinant of metastatic colonization (146–148).

The potential short- and long-term detrimental effects of different cancer treatments on monopoiesis and peripheral monocytes represents another important gap in our knowledge. Indeed, some reports suggest that surgery induces the mobilization and immunosuppressive reprogramming of circulating monocytes that may contribute to early metastatic relapse after tumor resection (149, 150). Thus, therapy-induced changes in monocytes and their role in therapy resistance as well as disease progression will be another relevant area of investigation in the future.

Overall, a deeper understanding of systemic monocyte reprogramming in cancer could not only lead to better clinical biomarkers but could also lead to novel therapeutic approaches with the potential to establish long-term antitumor immunity and prevent disease progression.

## Author Contributions

MK wrote the manuscript and designed the figures. AC edited the manuscript and designed the figures. GR and DL edited the manuscript. All authors contributed to the article and approved the submitted version.

## Conflict of Interest

The authors declare that the research was conducted in the absence of any commercial or financial relationships that could be construed as a potential conflict of interest.
